# Evaluation of an electronic psycho-oncological adaptive screening program (EPAS) with immediate patient feedback: findings from a German cluster intervention study

**DOI:** 10.1007/s11764-021-01121-8

**Published:** 2021-11-04

**Authors:** Peter Esser, Leon Sautier, Susanne Sarkar, Georgia Schilling, Carsten Bokemeyer, Uwe Koch, Matthias Rose, Michael Friedrich, Sandra Nolte, Otto Walter, Anja Mehnert-Theuerkauf

**Affiliations:** 1 Department of Medical Psychology and Medical Sociology, University of Leipzig, Leipzig, Germany; 2 Department of Medical Psychology, University Medical Center Hamburg-Eppendorf, Hamburg, Germany; 3 Clinical Psychology and Psychotherapy, Institute of Psychology, Faculty of Psychology and Movement Sciences, University of Hamburg, Hamburg, Germany; 4 “Hubertus Wald” Tumor Center, University Cancer Center Hamburg (UCCH), University Medical Center Hamburg-Eppendorf, Hamburg, Germany; 5 Department of Clinical Oncology, Asklepios Tumorzentrum Hamburg, Hamburg, Germany; 6 Department of Internal Medicine II, University Medical Center Hamburg-Eppendorf, Hamburg, Germany; 7 Medical Clinic, Department of Psychosomatic Medicine, Charité - Universitätsmedizin Berlin, Berlin, Germany

**Keywords:** Cancer, Psycho-oncology, Distress, Patient-reported outcomes, Quality of life, Screening

## Abstract

**Purpose:**

Distress screening has become mandatory and essential in comprehensive cancer care. We evaluated an electronic psycho-oncological adaptive screening (EPAS) which assesses objective indicators of care needs and subjectively perceived care needs and subsequently provides patient feedback with individualized recommendations about psychosocial care services.

**Methods:**

Patients were assessed within clusters, i.e., different oncological facilities of the competence network of the University Cancer Center Hamburg (UCCH). Patients in the intervention arm underwent the screening, controls received standard care. Patients were assessed at baseline (t0), 3-month (t1), and 6-month (t2) follow-up. Outcomes included information level and use of/access to nine psychosocial services at UCCH, well-being (GAD-7, PHQ-9, SF-8), and treatment satisfaction (SCCC). Conditional linear and logistic regressions were used to identify screening effects at t1 and t2.

**Results:**

Of 1320 eligible patients across 11 clusters, 660 were included (50%). The average age was 60 years; 46% were female. The intervention was associated with increased information level for all psychosocial services at t1 and t2 (all *p* < .001), increased use in some of these services at t1 and t2, respectively (*p* ≤ .02), and better evaluation of access (e.g., more recommendations for services provided by physicians, *p* < .01). At t2, the intervention was associated with a lower level of satisfaction with disease-related information (*p* = .02).

**Conclusions:**

EPAS may improve information about psychosocial services as well as utilization of and access to these services. The effect on information level seems not to be generalizable to other aspects of oncological care. Future studies should incorporate novel technologies and condense the procedure to its core factors.

**Implications for Cancer Survivors:** The screening may help to enhance self-management competencies among cancer survivors.

**Trial registration:**

The trial was retrospectively registered (2/2021) at ClinicalTrials.gov (number: NCT04749056).

**Supplementary Information:**

The online version contains supplementary material available at 10.1007/s11764-021-01121-8.

## Introduction

Due to multiple challenges in all areas of life, many cancer patients show elevated levels of mental burden such as depression or anxiety [1, 2]. Such symptomatology, conceptualized under the broader term *distress* [3], may worsen quality of life and even medical outcomes such as morbidity and mortality [4]. Nevertheless, many distressed patients are not recognized by the treating clinicians [5] and left untreated even though effective psychosocial interventions exist [6]. Therefore, screening for distress to detect those in need is considered mandatory in comprehensive cancer care [7]. In recent years, the general feasibility of electronic distress screenings in oncological routine care has been repeatedly demonstrated [8–12].

Previous findings on the effects of screenings on well-being, communication and referral are mixed, and thus the general benefit of screening is often argued [13]. Discrepant findings, however, may be caused by barriers that impede the usefulness of the screenings, such as lack of qualification of the physicians in interpreting results or a lack of transformation of screening results into individualized support plans [13–15]. Resolving such barriers requires extensive and repeated training [14, 16], which in turn may hamper the long-term effectiveness of a screening program. Therefore, reducing the burden for the medical staff to a minimum by facilitating referral to the psychosocial services within the respective institution may support long-term implementation of such a program.

Another important issue in screening is the time that patients need to complete the self-report questionnaires. Previous screenings mostly use conventional instruments based on classical test theory such as the Patient Health Questionnaire-9 (PHQ-9) [17] or the Generalized Anxiety Disorder-7 (GAD-7) [18] which present the same set of items to all participants. An alternative to these fixed-length questionnaires is the application of computerized-adaptive tests (CATs) based on item response theory that considers both item characteristics and the individual response pattern. CATs work in such way that they only present those items to individual respondents that are most relevant to them, thereby selecting an item subset from a larger pool of items (i.e., item bank). Such tailoring reduces respondent burden and thus may result in improved acceptability among patient populations and healthcare providers [19].

From a conceptual perspective, current distress screenings mostly rely on objective indicators of supportive care needs, i.e., they use patient-reported levels of distress to decide about further actions. This approach assumes that such objective indicators are closely linked to the subjectively perceived care need of a patient. However, recent research has revealed that objective indicators of care needs and subjectively perceived care needs are not necessarily related: For example, a study including a large population (*n* = 4020) demonstrated that only 51% of the patients with elevated distress levels reported a need for psychosocial support, whereas 26% of those without elevated distress levels reported such a need [20]. Therefore, both distress values as objective indicators and subjectively perceived care needs may be important to be included in distress screening programs.

We developed an electronic psycho-oncological adaptive screening program (EPAS) which incorporates both levels of distress and subjectively perceived care needs and subsequently provides immediate patient feedback with individualized recommendations about psychosocial care services at the care facility. We evaluated the screening by testing the effect of EPAS on all steps of the screening process, i.e., starting from information level about the psychosocial services up to mental health outcomes.

## Methods

### Study design and participants

Patients were assessed within clusters, i.e., different inpatient and outpatient cancer care facilities of the competence network of the University Cancer Center Hamburg (UCCH). Initially, we aimed to form matched pairs of clusters according to similar characteristics in order to allocate comparable clusters to the intervention and control condition, respectively. However, such a matching did not seem feasible due to differences in numbers and characteristics of patients across clusters. Therefore, we addressed the cluster bias by aiming to assess a similar number of patients in each cluster for each condition. To reduce the risk of any interfering effect between patients from different conditions, we prevented any overlap of conditions among patients that were in the same (waiting) room. This was accomplished by different measures, e.g., by suspending recruitment before changing the condition until the set of patients had completely changed or by assessing in spatially separated locations within the respective cluster.

Participants were eligible if they were (i) diagnosed with any malignancy according to ICD-10 irrespective of remission or treatment status or history of relapse, (ii) aged 18 years or older, (iii) able to read and speak German, and (iv) accessible for the research assistants (e.g., excluding those who were currently in isolation to prevent infection). For the intervention, patients needed to be skilled for using a tablet. Patients with any impairments interfering with the ability to give informed consent were excluded.

Patients were checked for eligibility via information provided by the treating physician and review of the medical chart. They were consecutively recruited by trained research assistants during their visit at the respective care facility for either treatment or medical check-up. To avoid repeated study invitation, the list of current patients in the respective cluster was compared with the list of previous participants before each recruitment. Each patient received only one condition; the intervention consisted of one screening.

The baseline assessment (t0) for the intervention group was completed using a tablet computer at the respective cancer care facility; during this session, both the measures of the intervention (EPAS) and the baseline study outcomes were assessed. The control group received a paper pencil questionnaire to be completed at the facility or at home using a pre-stamped envelope. At 3 months (t1) and 6 months (t2) follow-up, all participants were sent paper–pencil questionnaires by mail and reminded if the questionnaire were not returned within 2 weeks. The intervention was non-randomized and unblinded.

All participants provided written informed consent. The study protocol was approved by the ethics committee of the medical chamber of Hamburg (PV4371). The study was retrospectively registered (2/2021) at ClinicalTrials.gov (number: NCT04749056).

### Intervention condition (EPAS)

*Principle and procedure*: EPAS (electronic psycho-oncological adaptive screening) is a tablet-based screening application consisting of (i) three adaptive tests assessing levels of distress (= objective indicators of care needs) and (ii) one supportive care checklist to explicitly report the need for specific psychosocial services (= subjectively perceived care needs). EPAS provides immediate feedback via a printed results page, which presents and interprets the level of distress and contains individualized recommendations for psychosocial services. The results pages were printed by research assistants immediately after the screening on a mobile printer and given to the participants together with a brochure containing information about all psychosocial services available at the UCCH. The treating physicians received a slightly different results page, but were not expected to discuss these with the patient unless they were highly distressed (see Algorithm section). Before and during the screening, patients were explained how to use the program by the research assistant and were supported if needed.

*Measures within EPAS*: To assess levels of distress, CATs were applied, i.e., the depression CAT (D-CAT) to assess depression [21], the anxiety CAT (A-CAT) to assess anxiety [22], and stress CAT (S-CAT) to assess stress which was further divided into two separate CATs on stress exposure and stress reaction, respectively [23]. From the respective item bank, only items with the highest information value were selected according to both item characteristics and individual response pattern. The presentation of items ended if the standard error was ≤ 3.2 or a maximum of 10 items was reached. Completion time for each of the CAT instruments ranged from 96 s (D-CAT) to 151 s (A-CAT), the mean completion time for all 4 adaptive screening instruments was 8.3 min. The mean number of items ranged between 6 (D-CAT) and 10 (S-CAT, stress exposure), with corresponding standard errors of the respective theta values being 3.0 and 4.1, respectively. In addition to the CAT instruments, patients filled in an internally developed checklist to report supportive care needs across different topics, e.g., psycho-oncological or social counseling (Table S1).

*Structure and content of results page*: The results page contained (i) the extent of distress, (ii) a summary of the reported supportive care needs, and (iii) individualized recommendations for the use of psychosocial services at the UCCH based on the care needs. For all adaptive tests, aids to interpret the respective levels were provided: For the D-CAT and the A-CAT, categories of “low,” “medium,” and “high” distress were defined. For the D-CAT, these categories had been derived from a standardization study aimed at defining a common metric for depression based on data from psychosomatic patients and the general population [24]. For the A-CAT, no norm data were available: Therefore, we selected patients from a psychosomatic sample that had completed the GAD-7 [18] alongside the A-CAT (*unpublished data*). In doing so, we were able to determine the respective theta values that corresponded to the GAD-7 scores of 5 (low), 10 (medium), and 15 (high). In contrast, no data were available as a gold standard for assigning severity categories to the S-CAT: To facilitate data interpretation, we instead used a patient sample diagnosed with either adjustment disorder or burn-out syndrome and applied the respective mean (separately for stress reaction and stress exposure) as a reference value (*unpublished data*). Of note, the patient and physician versions of the feedback page differed slightly: Patients were explicitly referred to the information brochure which they received during the screening. In contrast, the distress categories in the physician version were illustrated with colors (low = green; medium = yellow; high = red) and contained specific information for highly distressed patients (see Algorithm section).

*Algorithm for results page*: The supportive care needs reported in the checklist were transformed into concrete recommendations for using the adequate psychosocial service at the UCCH (e.g., a reported need for support in the topic “return to work” resulted in a recommendation to use the social service; Table S1). Highly distressed patients, i.e., those falling into the category “high” in the A-CAT or D-CAT, were recommended to use psycho-oncological service irrespective of whether they had reported such a need in the checklist. The treating physicians of highly distressed patients were recommended on their results page to talk with the patient about his/her psychosocial condition and were given further suggestions for such an appointment (e.g., check for medical reasons for distress, encourage patients to use psycho-oncological service).

### Control condition

Controls completed all three assessments (i.e., at t0, t1, and t2) via paper pencil questionnaires. From the measures used in the EPAS intervention (CATs and supportive care checklist), the controls completed a paper pencil version of the supportive care checklist, but did not complete CATs which require an electronic assessment. Neither patients nor physicians received any feedback of the results. Psychosocial services were recommended by the physicians on their own discretion only. Patients did not receive the information brochure. Nevertheless, patients had the same access to all psychosocial services as the intervention group, and the information brochure was visible and available at all centers of the UCCH.

### Outcomes

Since this screening approach had not been tested before, we were equally interested in effects of the screening across all levels of psychosocial care, i.e., from being informed about psychosocial services up to their potential benefits for mental health. Therefore, we decided against hierarchization in primary and secondary outcomes. Nevertheless, we note that the outcome *information level* was considered the basic requirement for all other outcomes and thus may represent the central outcome.

#### Information level about psychosocial services

We internally developed single item scales assessing the level of information for each of the psychosocial services which were available at the UCCH. On a five-point Likert scale ranging from 0 (“I do not even know that such a program exists”) to 4 (“very well”), patients rated how well they feel informed about the respective service.

#### Use of psychosocial services/evaluation of access

Analogously to the single item scales to assess information level, we internally developed binary items to assess the respective *use of the psychosocial services* available at the UCCH (yes/no).

To evaluate the access to the services, we internally developed binary items (yes/no) to assess whether any (i) communication with the physicians about supportive/complementary care needs, (ii) recommendations by the physicians for specific psychosocial services, (iii) concrete offers by the physicians to use a psychosocial service, or (iv) request by the physicians for a consultation service had taken place.

#### Well-being and treatment satisfaction

All of these outcomes were assessed in both the intervention and control group using validated questionnaires. In detail, well-being was assessed via depression (PHQ-9 [17]), anxiety (GAD-7 [18]), and quality of life (SF-8 [25]), while treatment satisfaction was assessed via the Satisfaction with Comprehensive Cancer Care (SCCC) questionnaire [26].

#### Sociodemographic and medical data

Sociodemographic and medical data were obtained from self-report and medical chart, respectively.

#### Administration of outcomes

Since more than half of the patients were diagnosed very recently (≤ 3 months), care relevant outcomes, i.e., items related to the psychosocial services at the UCCH and treatment satisfaction, were not expected to be applicable at t0 and thus were assessed at t1 and t2 only. Variables on the evaluation of access to the services were assessed once (t1). Variables on well-being (depression, anxiety, and quality of life) were assessed at all measurement points. Given that the other outcomes were not assessed at t0, we did not include baseline scores of the well-being variables in the respective analyses in order to use the same covariates for each analysis and thus to ensure comparability of the findings.

### Power analysis

The study aimed to test for group differences between conditions, separately for t1 and t2. To detect an expected small to medium group difference in level of information (effect size = 0.3) with a power of 80%, sample sizes of 176 patients in each group were needed. We initially expected a drop-out rate of 30% until t2, resulting in a minimum of *n* = 251 per group (*n*_total_ = 502).

### Statistical analyses

We provided descriptive statistics for sociodemographic and medical data. To investigate baseline differences between conditions, comparisons were conducted via logistic regression and *t* tests for binary and continuous data, respectively. The same tests were applied to investigate representativeness of the sample at the follow-up time points by comparing drop-outs vs. study completers.

To investigate the group effect of the intervention, we applied linear (information level/well-being/treatment satisfaction) and logistic (use of service/evaluation of access) regression analyses, separately for t1 and t2.

All models were controlled by covariates. Given that group effects were investigated for both t1 and t2, an empirical approach to select covariates according to differences between conditions or between drop-outs and completers would have resulted in different covariates at both measurement points and thus would have limited comparability of the findings. Therefore, covariates were selected based on theory. In detail, we selected the two variables which were supposed to be particularly important for care relevant outcomes [20] and were also used as the two domains within the EPAS, i.e., objective indicators of care need (= level of general distress measured with the distress thermometer (DT) [27] at t0) and subjectively perceived care need (= percentage of reported needs on the supportive care checklist at t0). Additionally, we included gender and age as central sociodemographic variables. Covariates were checked for multicollinearity (bivariate correlations *r* < 0.7).

For the linear regression models, we also provide *ΔR*^*2*^ to indicate the change in explained variance of the model after having added the intervention variable (calculation: *R*^2^_whole model_—*R*^2^_model without intervention variable_). For the logistic regressions, we report odds ratios (OR): values > ( <) 1 mean that the odds to experience the respective event in the intervention group are higher (lower) than in the control group. For example, an OR = 4 means that for every 4 persons that experience the event in the intervention group, 1 person will experience the event in the control group [28]. The alpha level was set at 0.05. Listwise deletion was applied; analyses were conducted using SPSS (Version 25).

### Deviations from initial study proposal

Given the retrospective trial registration, we attached a translation of the synopsis of the initial study proposal to ensure maximum transparency (Table S2). Any points by which the final report of the study deviates from the initial study proposal are listed and explained in Table S3.

## Results

### Participant flow

**Fig. 1:**
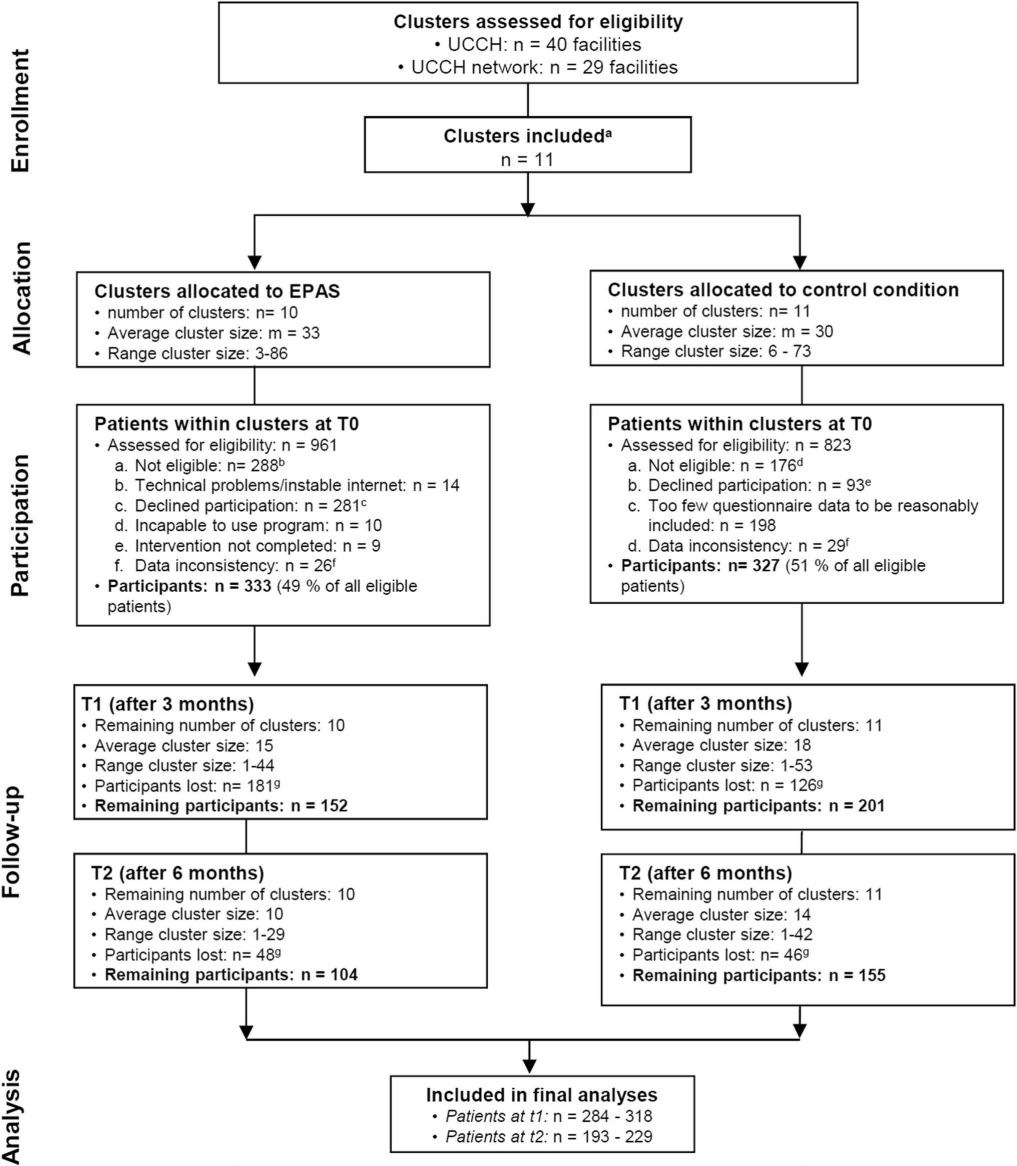
Flow chart. UCCH, University Cancer Center Hamburg. ^a^Clusters were included if they (i) were primary oncology facilities, (ii) treating a high number of patients, and (iii) agreed to participate in the study; each cluster received each condition except for the cluster “Marienkrankenhaus—Private station” which only received control condition owing to too few participants. ^b^Severe physical/mental/cognitive impairment (*n* = 193), isolated (*n* = 18), insufficient language skills (*n* = 68), incompetence to use tablet as assessed by physicians (*n* = 9). ^c^Physical/mental burden (*n* = 32), organizational issues (*n* = 19), no interest (*n* = 171), other reasons (*n* = 59). ^d^Severe physical/mental/cognitive impairment (*n* = 130), isolated (*n* = 21), insufficient language skills (*n* = 23), underage (*n* = 2). ^e^Physical/mental burden (*n* = 14), organizational issues (*n* = 8), no interest (*n* = 66), other reasons (*n* = 5). ^f^Within clearance of the final data, any cases with unrestorable documentation errors or missing/unclear information on either age, gender, or diagnosis were deleted. ^g^Patient loss mostly due to not sending back the questionnaires or having deceased

We recruited from December 2013 to December 2014; data collection was completed in July 2015. Eleven clusters participated in the study, among which 1784 patients were checked for eligibility (Fig. 1). In the intervention arm, 333 out of 673 eligible patients were included (49%). In the control arm, 327 out of eligible 647 patients were included (51%). The drop-out rate until t2 was 69% and 53% for the intervention and the control condition, respectively. For additional information on included clusters, see Table S4.

Compared to study completers, drop outs were more likely to be in the intervention group (*p* < 0.001) and to have metastases (*p* = 0.03), palliative treatment (*p* < 0.001), and a history of relapse (*p* = 0.01). They were less likely to have a partner (*p* = 0.03) or a hematological cancer diagnosis (*p* = 0.001) and had a longer time since diagnosis (*p* = 0.04).

### Baseline sample characteristics

The average age was 60 years, 46% were female (Table 1). The most frequent cancer types were hematological and colorectal (32 and 12%, respectively), and more than half or participants were diagnosed within the last 3 months.

**Table 1:** Baseline sample characteristics *(if not else noted: raw values, valid percentages in parentheses)*

		Total (*N* = 660)	EPAS (*n* = 333)	Control (*n* = 327)	
		*n* %	*n* %	*n* %	*p*^i^
Age in years (M, SD)		60 (14)	57 (14)	62 (13)	< .001
Gender	Female	300 (46)	159 (48)	141 (43)	.23
Partnership	Yes	363 (59)	140 (46)	223 (72)	< .001
Education	≤ 10 years at school	176 (28)	60 (20)	116 (36)	< .001
	> 10 years at school	443 (72)	237 (80)	206 (64)	
Cancer type (ICD-10)	Hematologic^a^ (C81-86, C90-93, C95, D46)	211 (32)	111 (33)	100 (31)	.45
	Colorectal (C17-21)	77 (12)	29 (9)	48 (15)	
	Lung (C34)	68 (10)	28 (8)	40 (12)	
	Breast (C50)	68 (10)	31 (11)	32 (10)	
	Head and neck (C00-02, 04, 07–11, 13/14, 30, 32, 76)	35 (5)	17 (5)	18 (6)	
	Stomach/Eosophagus (C15/16)	30 (5)	12 (4)	18 (6)	
	Prostate (C61)	25 (4)	12 (4)	13 (4)	
	Pancreas (C25)	23 (4)	11 (3)	12 (4)	
	Gall/liver (C22, 24)	15 (2)	7 (2)	8 (2)	
	Female genital organs (C53/54, 56/57)	17 (3)	12 (4)	5 (2)	
	Urogenital (C64, 67/68)	13 (2)	10 (3)	3 (1)	
	Testis (C62)	13 (2)	8 (2)	5 (2)	
	Others^b^	65 (10)	40 (12)	25 (8)	
Treatment setting	Outpatient	375 (57)	172 (52)	203 (62)	.01
	Inpatient	285 (43)	161 (48)	124 (38)	
Months since current diagnosis (M, SD)		12 (27)	11 (28)	13 (26)	.48
	≤ 3 months	337 (52)	170 (52)	167 (52)	
	> 3 months	312 (48)	160 (49)	152 (48)	
TNM-T^c^	0: no tumor/CUP	7 (2)	4 (2)	3 (2)	.44
	1: < 2 cm	43 (11)	23 (12)	20 (10)	
	2: 2–5 cm	73 (19)	29 (16)	44 (22)	
	3: > 5 cm	119 (31)	59 (32)	18 (30)	
	4: ext. to skin/chest wall	61 (16)	31 (17)	30 (15)	
	X: not assessable	82 (21)	40 (22)	42 (21)	
TNM-N^d^	0: no lymph node metastases	94 (24)	47 (25)	47 (24)	.53
	1–3: lymph node metastases	189 (49)	87 (47)	102 (51)	
	X: not assessable	102 (27)	52 (28)	50 (25)	
TNM-M^e^	0: no distant metastases	147 (38)	75 (40)	72 (36)	.40
	1: distant metastases	236 (61)	110 (59)	126 (63)	
	X: no information	2 (1)	1 (1)	1 (1)	
UICC stadium^f^	I	25 (6)	13 (6)	12 (5)	.60
	II	41 (9)	21 (10)	20 (9)	
	III	76 (17)	38 (18)	38 (17)	
	IV	243 (56)	115 (54)	128 (57)	
	Not evaluable	52 (12)	26 (12)	26 (12)	
Relapse	Yes	170 (26)	94 (28)	76 (24)	.17
Type of treatment^g^	Surgery	255 (39)	153 (46)	102 (31)	< .001
	Radiation	176 (27)	107 (32)	69 (21)	.002
	Chemotherapy	591 (90)	294 (89)	297 (91)	.23
Treatment intention	Curative	280 (46)	151 (50)	129 (42)	.06
	Palliative	328 (54)	152 (50)	176 (58)	
Karnofsky index (M, SD)		96 (9)	96 (10)	95 (9)	.30
General distress (M, SD)		5 (2)	4.7 (2)	5.3 (2)	.001
Supportive care need (M, SD)^h^		40 (25)	42 (24)	39 (26)	.11

Note: percentages may not add up to 100 due to rounding

*SD*, standard deviation

^a^Mostly non-Hodgkin lymphoma (*n* = 94) and multiple myeloma (*n* = 43); ^b^ICD-10: C7A, 40/41, 44/45, 48/49, 70/71, 74/75, 78, 80; ^c^tumor staging via size and extent of the tumor, excluding hematological cancer; ^d^tumor staging via degree of spread to regional lymph nodes, excluding hematological cancer; ^e^tumor staging via presence of distant metastasis, excluding hematological cancer; ^f^excluding hematological cancer; ^g^current or completed; ^h^percentage of reported care needs within the checklist (range 0–100); ^i^comparison between intervention and control group via *t* tests and logistic regressions; nominal data were dummy coded for analyses (cancer type: hematological vs. non-hematological; TNM-T: 1–2 vs. 3–4; TNM-N: 0 vs. 1–3; TNM-M: 0 vs. 1; UICC: I–II vs. III–IV)

Compared to the controls, patients in the intervention group were younger, better educated, less distressed, more likely to be inpatient and to be treated with surgery and radiation, but less likely to be in a relationship (Table 1).

### Effect on level of information

At both t1 and t2, the intervention was significantly associated with a higher level of information across all nine psychosocial services (Table 2). *ΔR*^*2*^ indicated that the intervention variable increased the explained variance of the whole model with up to 23% (*cancer survivorship program* at t1).

**Table 2:** Group effect of the screening on level of information about psychosocial services, controlled for gender, as well as baseline age, distress, and level of supportive care need

	*N*	*B (SE)*	*p*	*ΔR*^*2*^
**T1 (3 months follow-up)**				
Social service	309	.84 (.13)	< .001	.11
Psycho-oncology	306	.87 (.14)	< .001	.11
Nutritional advice	304	.73 (.14)	< .001	.08
COSIP^a^	284	1.2 (.13)	< .001	.22
Complementary medical lesson	305	1.3 (.12)	< .001	.25
Activity and sport programs	308	1.0 (.13)	< .001	.16
Palliative consultation	307	1.1 (.13)	< .001	.18
Prevention counselling	307	1.2 (.13)	< .001	.22
LOTSE (cancer survivorship program)	307	1.3 (.13)	< .001	.23
**T2 (6 months follow-up**)				
Social service	221	.71 (.15)	< .001	.08
Psycho-oncology	216	.76 (.16)	< .001	.09
Nutritional advice	216	.82 (.15)	< .001	.12
COSIP^a^	193	.94 (.16)	< .001	.15
Complementary medical lesson	219	.75 (.15)	< .001	.11
Activity and sport programs	219	.82 (.14)	< .001	.13
Palliative consultation	215	.74 (.15)	< .001	.10
Prevention counselling	218	.86 (.14)	< .001	.14
LOTSE (cancer survivorship program)	219	.90 (.13)	< .001	.17

*B*, unstandardized regression coefficient: positive values indicate that the intervention is associated with an increase in the respective information level; *SE*, standard error; *ΔR*^*2*^, change in explained variance R^2^ after having added the intervention variable to the model

^a^Program for children with parents with cancer

### Effect on use of psychosocial services and the evaluation of access

At t1 and t2, the intervention was significantly associated with more frequent use of the *activity and sports programs* (Table 3). Furthermore, the intervention was significantly associated with more frequent use of the service *complementary medical lesson* at t2. The OR of the significant effects reached up to 2.2 and 4.1 at t1 and t2, respectively.

**Table 3:** Group effect of the screening on use of psychosocial services and evaluation of access, controlled for gender as well as baseline age, distress, and level of supportive care need

	*N*	*B (SE)*	*p*	*OR [95% CI]*
**T1 (3 months follow-up)**				
*Use of services*				
Social service	313	.24 (.27)	.37	1.27 [.75;2.2]
Psycho-oncology	315	.33 (.29)	.26	1.39 [.78;2.4]
Nutritional advice	309	.26 (.29)	.37	1.30 [.73;2.3]
COSIP^a^	305	− .90 (1.2)	.45	.41 [.04;4.2]
Complementary medical lesson	308	.85 (.47)	.07	2.3 [.94;5.8]
Activity and sport programs	310	.80 (.33)	.02	2.2 [1.2;4.3]
Palliative consultation	303	.24 (.44)	.58	1.3 [.54;3.0]
Prevention counselling	304	.68 (.64)	.29	2.0 [.56;7.0]
LOTSE (cancer survivorship program)	305	1.2 (.87)	.17	3.3 [.60;17.9]
*Evaluation of access*				
Talk about services	302	.88 (.25)	< .01	2.4 [1.5;4.0]
Recommendation for services	295	.73 (.25)	< .01	2.1 [1.3;3.4]
Offering consultation	298	.67 (.26)	.01	2.0 [1.2;3.3]
Request of consultation	290	.06 (.27)	.82	1.1 [.63;1.8]
**T2 (6 months follow-up)**				
*Use of services*				
Social service	229	.44 (.32)	.18	1.5 [.82;2.9]
Psycho-oncology	228	.66 (.36)	.07	1.9 [.94;3.9]
Nutritional advice	223	.64 (.34)	.06	1.9 [.97;3.7]
COSIP^a^	215	1.4 (1.7)	.42	3.9 [.14;107]
Complementary medical lesson	224	1.4 (.59)	.02	4.1 [1.3;12.9]
Activity and sport programs	227	1.2 (.40)	< .01	3.3 [1.5;7.2]
Palliative consultation	225	.52 (.59)	.38	1.68 [.53;5.3]
Prevention counselling	226	.45 (.65)	.49	1.6 [.44;5.6]
LOTSE (cancer survivorship program)	227	1.2 (1.3)	.35	3.2 [.27;38.6]

*B*, unstandardized regression coefficient: positive (negative) values indicate that the intervention is associated with an increase (decrease) in the likelihood of an occurrence of the respective event; *OR*, odds ratio: a value > ( <) 1 means that the odds to experience the respective event in the intervention group are higher (lower) than in the control group; *SE*, standard error; *p*, *p* value related to the regression coefficient based on the Wald statistic; *95% CI*, 95% confidence interval

^a^Program for children with parents with cancer

The intervention was associated with better evaluation regarding the access of the services. In detail, patients more frequently talked with their physician about supportive care services, received more recommendations on such services, and were more often given concrete offers to use these psychosocial services. The OR of the significant effects ranged from 2.0 to 2.4.

### Effect on well-being and patient satisfaction

The intervention variable was not significantly associated with any measures of well-being (Table 4). Patients in the intervention group were significantly less satisfied with their level regarding disease-related information; however, the intervention variable only added 2% of explained variance to this overall model effect.

**Table 4:** Group effect of the screening on well-being and treatment satisfaction, controlled for gender as well as baseline age, distress, and level of supportive care need

	*N*	*B (SE)*	*p*	*ΔR*^*2*^
**T1 (3 months follow-up)**				
*Well-being*				
Depressive symptoms	318	.08 (.54)	.89	< .001
Anxiety	318	.18 (.43)	.68	< .001
Mental quality of life	311	.51 (.97)	.60	.001
Physical quality of life	311	− .92 (.84)	.28	.004
*Treatment satisfaction*				
Competence	314	− .07 (.07)	.33	.003
Information	314	− .07 (.07)	.32	.003
Access	259	.06 (.10)	.60	.001
Support	298	− .02 (.08)	.79	< .001
Overall medical treatment	313	− .01 (.08)	.87	< .001
Overall psychosocial treatment	290	.02 (.15)	.91	< .001
**T2 (6 months follow-up)**				
*Well-being*				
Depressive symptoms	225	− .02 (.60)	.97	< .001
Anxiety	225	.02 (.50)	.97	< .001
Mental quality of life	225	.69 (1.0)	.50	.002
Physical quality of life	225	− .18 (1.1)	.87	< .001
*Treatment satisfaction*				
Competence	222	− .10 (.08)	.22	.006
Information	221	− .22 (.09)	.02	.02
Access	206	− .17 (.12)	.17	.009
Support	218	− .10 (.09)	.24	.005
Overall medical treatment	222	− .12 (.09)	.21	.007
Overall psychosocial treatment	222	− .02 (.19)	.92	< .001

B, unstandardized regression coefficient: positive (negative) values indicate that the intervention is associated with an increase (decrease) in the respective outcome; SE, standard error; *ΔR*^*2*^, change in explained variance *R*^2^ after having added the intervention variable to the model

## Discussion

### Main findings

This cluster intervention showed that an electronic psycho-oncological screening with immediate patient feedback was associated with an increased level of information about different psychosocial services, better support by physicians to access these services, and increased use in one and two of these services after 3 and 6 months, respectively.

### Integration into previous research

Comparability with previous studies is limited: Previous studies on online distress screenings mostly tested feasibility, but did not assess the effect on specific outcomes, e.g., [8–12, 29]. Furthermore, we identified self-management programs with tailored patient feedback among recent reviews (*n* = 12 [30] and *n* = 13 [31]), but these programs primarily addressed medical management and/or consisted of several assessments. A recent RCT (*n* = 625 survivors) on an online screening with subsequent individualized feedback on supportive care options was conceptually similar to ours [32]. Nevertheless, they used different outcomes (primary outcome: patient activation) and thus is hard to compare with our findings.

We found large effects of the screening on information level across all types of psychosocial services. Since information level forms the basis to empower patients in using support, this result is central for our initial aim to improve health-related self-management. Several aspects within the screening may have contributed to this effect, including the communication with the study assistants or the provision of the information brochure. For example, additional analyses (*data not shown*) revealed a strong positive intervention effect on the degree to which the brochure had been read. In addition, more than half of the patients in the control condition reported that they did not even know that such an information brochure existed even though brochures were available throughout the facilities. Such findings indicate that future research is needed to condense the screening procedure to its core elements, e.g., determine the extent by which the information level could be achieved by handing out the brochure alone.

The screening was associated with a more frequent use in some of the psychosocial services. The effect may be partly explained by the improved support by physicians in accessing these services in terms of more frequent communication, recommendation, and referral to consultation-liaison services. The higher referral rate which resulted from the screening is in line with a recent screening intervention using a stepped-care approach [16]. The OR reached up to 4 (service *complementary medical lesson* after 6 months), meaning that for every 4 people that used this specific service in the intervention group, only one person used this service in the control group. For most services, however, we did not find an effect: As an explanation, the benefit may depend on patient characteristics such as self-efficacy or health literacy [33] and thus respective effects may only be found for certain subgroups of patients. As a second explanation, it is to note that patients were relatively shortly after their diagnosis: It is possible that patients need time to adjust to the new situation, to recognize their need for supportive care, and finally to decide for any professional support. Since OR may be overestimated in small sample sizes [34], we note that the comparability of effects between time points is limited given different sample sizes.

In line with previous research, we did not find any screening effect on well-being [16, 35–38]. One explanation may be a floor effect. For example, one of the few studies (*n* = 116 breast cancer patients) which found a significant improvement in anxiety and depression pre-selected patients with high distress (DT ≥ 7) [39], which was only true for 31% of the patients in our study (*data not shown*). Furthermore, the study design might not have been adequate to detect effects on well-being: Given that we could not control whether and which psychosocial service was actually used and that the control group had the same access to the services, an equal intervention effect related to these outcomes could not be investigated.

The screening was associated with a lower level of satisfaction with disease-related information, which may first seem contra-intuitive. Two explanations, however, seem reasonable: First, this 7-item scale assessed mainly medical information, with only one item assessing the information level related to psychosocial services; therefore, our screening was not adequate to improve this specific outcome. Second, patients in the intervention group may have used their elevated level of information regarding psychosocial services as a reference for “satisfying information” and thus may have been “stricter” in their evaluation of the information level with medical aspects.

### Implications

In light of the lack of a direct effect on well-being, some authors argue if an implementation of such a program is warranted [40]. However, we think that such a decision needs to consider both efforts and benefits: In the current approach, we kept the effort of the medical staff as low as possible. This in turn may help to reduce institutional implementation barriers such as resources, training/education, or organizational readiness [41, 42]. Furthermore, we found robust effects in strengthening patient autonomy through information and improving awareness in physicians. Therefore, the benefits may speak in favor for the implementation of such a program.

Several adaptations may further optimize the screening. First, the project started in 2013 and thus the program needs to be adapted to the new technological developments and made applicable on mobile devices such as smartphones. In doing so, a specific app may guide the patient through the program, and the results might be directly sent to the patients and physicians which would further improve efficiency. Detailed assessment of the number and extent of help provided by the research team in using such apps will help to improve usability. For institutions with fewer psychosocial services, the algorithm needs to be adapted, e.g., by referring patients to online support services, websites of ambulatory psycho-oncologists, or specific self-help groups. Given that the screening procedure consisted of several aspects, it remains unclear which elements are central for the respective benefits of the screening. Future studies need to identify such core elements to improve its efficiency. Secondary analyses may investigate moderator effects such as time since diagnosis or remission status to identify subgroups for which the screening is particularly effective.

### Strengths

We provided an innovative approach by combining several aspects such as (i) focus on psycho-oncological outcomes, (ii) use of adaptive screening instruments, (iii) assessing both levels of distress and subjectively perceived care needs, and (iv) making use of already existing psychosocial services at the healthcare facility. In doing so, we tried to optimize efficiency for all stakeholders which may increase the likelihood of the screening to be persistently applied in routine care settings. Given these strengths, our study including eleven clusters in different clinics and cancer care facilities may provide a novel approach for health services research in oncology.

### Limitations

We had a moderate response rate. As an explanation, patients had a mean age of 60, and thus a part of the eligible patients may not have been used to deal with digital devices back in 2013. It is also to note that patients were approached during their visit in the facility for treatment or medical check-up and thus they may not have been responsive for study participation. Among responders, the drop-out rate was relatively high, which may be caused by two issues: First, the majority of the sample was in a severe medical condition, with more than half being in palliative treatment. In fact, dropouts were more likely to have a worse physical status at baseline (i.e., more often metastases and relapse), which implies that many patients did not continue with the study because they felt too burdened to complete the assessments. Second, the questionnaires were relatively long and may have reduced motivation to complete the study: This assumption is supported by the fact that we observed a higher number of drop-outs in the intervention condition, for which the baseline assessment including the screening was longer than in the control group. Given that both conditions were applied in the clusters, we cannot rule out a carry-over effect among treating physicians in that they had become more aware of psychosocial issues in treating the control group after having started in the intervention condition. Even though we recruited more than patients than originally planned, we did not reach the minimum sample size in the final analyses, which may have limited test power. We also note that the outcomes on the psychosocial services were internally developed and thus might have lacked sensitivity. Finally, the trial was retrospectively registered. To ensure maximum transparency, however, we attached a translated version of the synopsis of the initial study proposal and provided details and reasons for any deviations.

### Conclusions

This study demonstrated that an electronic adaptive screening program for cancer survivors may enhance patient autonomy by increasing their information level and the use of psychosocial services and may improve the support by physicians to access these services. Future studies are needed to explore its use on mobile devices and to reduce the procedure to its core factors.

## Supplementary Information

Below is the link to the electronic supplementary material.

Supplementary file1 (DOCX 26 KB)

## Data Availability

The data and the code that support the findings of this study are available upon request from the authors.
