# Supplementary material for: Evaluation of an electronic psycho-oncological adaptive screening program (EPAS) with immediate patient feedback: findings from a German cluster intervention study
Source: J Cancer Surviv. 2021 Nov 4;16(6):1401–13. doi: 10.1007/s11764-021-01121-8 (PMC9630178; doi:10.1007/s11764-021-01121-8)
Supplement: Supplementary file 1 — Supplementary file1 (DOCX 26 KB) [file 11764_2021_1121_MOESM1_ESM.docx]

| **Table S1.** *Checklist for supportive care needs and their respective assignment to the psychosocial care services at the UCCH* | |
| --- | --- |
| **Items assessing specific supportive care needs** | **Assigned psychosocial service at the UCCH** |
| - Do you wish support by a psychologist/psycho-oncologist to deal with mental problems such as anxiety, despair or sadness? - Do you wish support by psychological/psycho-oncological assistance across all phases of the disease and its treatment? - Do you wish support by a psychologist/psycho-oncologist to deal with questions on existence and meaning? - Are you interested in information to foster your self-help strategies and to improve communication with close persons? | **Psycho-oncology** |
| - Are you interested in information regarding wage-replacement benefits, questions on current employment and/or return to work? - Are you interested in information how to issue a power of attorney or patient decree? - Are you interested in information how to get care and utility services? - Do you wish support in legal and regulatory issues such as questions related to fee providers and other relevant institutions? - Do you wish advice about the possibility of a subsequent cure such as rehabilitation? - Do you wish support in the search for a psychotherapist? - Do you wish support through participation in a self-help group? | **Social counseling** |
| - Do you wish advice and support as being a parent of underage children (e.g., how to talk to the child about the disease)? | **Program for children with sick parents (COSIP)** |
| - Do you wish counseling regarding optimal nutrition during and after the cancer treatment (e.g. keep or reach a healthy weight during chemotherapy)? - Do you wish advice on strategies how to cope with treatment-related loss of appetite and changes of taste? | **Nutritional advice** |
| - Are you interested in information about alternative and complementary treatment options (e.g., naturopathic or homeopathic)? | **Complementary medical lesson** |
| - Do you wish support with respect to activity and sports programs to maintain your physical fitness during and after treatment? - Do you wish support with respect to activity and sports programs in order to strengthen your immune defense and to reduce treatment-related side effects such as fatigue, pain and nausea? | **Activity and sports program** |
| - Are you interested in information about support options how to deal with an untreatable cancer disease (such as nursing and assistance, pain management, arrangement of hospice services and bereavement support groups, support for relatives)? | **Palliative consultation** |
| - Are you interested in information on possibilities to prevent the (re-) occurrence of cancer (prevention for both patients and healthy persons)? | **Consultation for cancer prevention** |
| - Are you interested in information about potential long-term and late effects of the disease and its treatment? - Are you interested in information with respect to medical follow-up care after your treatment has ended (e.g. the interval between and types of follow-up examinations)? | **Cancer survivorship program (LOTSE)** |

| **Table S2.** *Translated synopsis submitted within initial proposal (NKP-332-058)* | |
| --- | --- |
| **Applicant/principle investigator** | Anja Mehnert, PhD  Institute of Medical Psychology  University Medical Center Hamburg-Eppendorf (UKE) |
| **Project title /**  **ACRONYM** | Evaluation of an electronic psycho-oncological adaptive screening program to assess mental burden and psychosocial supportive care need in cancer patients/**EPAS** |
| **Area of research** | **Area of research 2/2.2.2:** Psychosocial/psycho-oncological support of cancer patients: comparison of models to inform about and provide psycho-oncological support |
| **Research aim /**  **hypotheses** | - evaluation of feasibility as well as testing of EPAS to asses mental burden and psychosocial supportive care needs in cancer patients and their relatives including the provision of evidence-based information, recommendation and referral to psychosocial support services - main research question: can EPAS contribute to higher level of information about psychosocial services, higher treatment satisfaction and competence and to reduced mental burden and improved quality of life in both patients and relatives? - hypotheses: EPAS significantly improves the level of information about psychosocial services, treatment satisfaction and competence in both patients and relatives |
| **Study design and methodological approach** | - quantitative and representative (including both in- and outpatients) cross-sectional assessment among cancer patients - comparative evaluation study: evaluation of EPAS within a controlled prospective intervention study, with care as usual as control condition (parallel group comparison based on quantitative longitudinally assessed patient data and cross-sectionally assessed physician data) |
| **Data base** | - Data obtained from patient surveys - Comparative evaluation: Assessment of patients (longitudinal) and physicians (cross-sectional) |
| **Sample** | - Patients across tumor entities (primary diagnosis/relapse/second cancer) treated in the competence network of the UCCH as in- or outpatients - Relatives of patients (explorative data assessment) - Physicians working in the oncologic health care facilities of the UCCH network |
| **Sample sizes** | - Cross-sectional assessment: 100 inpatients and 200 outpatients - Longitudinal assessment: 251 patients in each group (intervention and control) - Cross-sectional assessment of physicians: 40 to 50 physicians |
| **Analysis of data** | **Primary outcomes:**   - Level of information about psychosocial services, treatment satisfaction - Patient competence, mental burden and quality of life   **Secondary outcomes:**   - Acceptability and practicability of EPAS, evaluation of access to and use of psychosocial services, barriers and supporting factors to use psychosocial services   **Statistical Analyses:** descriptive, uni-/multivariate analyses |
| **Benefits und application possibilities of results** | Optimizing oncological treatment through higher patient competence and improved collaboration between patient and treating physician |
| **Involved institutions/**  **cooperation partners** | Institut für Medizinische Psychologie des UKE  Hubertus Wald-Tumorzentrum Universitäres Cancer Center Hamburg  Institut für Biometrie und Epidemiologie, UKE  Hämatologisch-Onkologische Praxis Altona (HOPA)  Onkologische Schwerpunktpraxis, Standort Ballindamm  Zertifiziertes Onkologisches Zentrum am Marienkrankenhaus |
| ***Please note:*** *Besides the longitudinal intervention study, the project included a cross-sectional pilot study. This part, however, is not relevant for the current article and thus is presented in grey font.* | |

| **Table S3.** *Deviations between final report and initial study proposal* | |
| --- | --- |
| **Domain** | **Type and rationale of deviation** |
| *Sample size* | - Given an unexpectedly high drop-out rate, we recruited more patients as originally planned (initial plan: n = 251 per group). |
| *Study design* | - We initially planned to match participating clusters according to similar patient characteristics to compensate for potential sample bias. Clusters from these matching pairs were planned to be assigned to either the intervention or control condition. Given that such a matching did not seem feasible due to large differences in numbers and characteristics of patients, we finally decided to assess a similar number of patients for each condition in each cluster. |
| *Selection criteria* | - We added the inclusion criteria “reachability” given that some patients were isolated and thus could not be contacted. - For the intervention group, we added the inclusion criteria “self-reported digital competence to use a tablet”. |
| *Measures within EPAS* | - The supportive care checklist initially contained 24 items: Due to technical problems, however, 3 items assessing the need for (i) creative therapy, (ii) advice and support for underaged child and (iii) second medical opinion were only assessed in one of the two conditions. Therefore, the final list that was used to assess subjective care needs and as covariate for the conditional regression models contained 21 items. |
| *Outcomes* | - We decided against a hierarchization in primary and secondary outcomes for this current article given that this approach was novel and thus we were equally interested in all different steps of the screening process, i.e., from being informed about psychosocial services until their potential benefits for mental health. - The 2 outcomes on feasibility of the screening (*acceptance* and *practicability*) and open questions on *barriers/supporting factors* to access psychosocial services were not reported in the current article given that this article should focus on effects of the screening compared to a control condition. - The outcome *patient competence* was assessed via personal resources (SWOP) and coping mechanisms (Mini Mac). Since both instruments measure relatively stable personality traits, these outcomes were not supposed to provide any interpretable findings about the effect of the screening after 3 or 6 months. Therefore, they were not included as outcomes in the final report. - The measure on general distress (DT) was chosen as a covariate in all analyses to control for baseline distress. Therefore, it was not used as an outcome in the final report. - Originally, the instruments assessing information level and use of the psychosocial services at the UCCH contained a 10th psychosocial service at the UCCH, i.e., “creative therapy”. Due to technical error in the supportive care checklist (see above), the only item assessing the need for this service was not assessed in the intervention group. Therefore, the outcomes related to “creative therapy” in each instrument could not be reasonably interpreted and thus respective findings were not reported. |
| *Exploratory analyses in relatives/*  *physicians* | - Originally, accompanying relatives of the patients should also be asked to participate in the screening program. However, no reference values of this population to interpret their emotional distress was available. Furthermore, the program developed for the patients turned out not to be applicable among relatives. Therefore, relatives were not included in the study. - A subgroup of physicians treating patients in the intervention group were asked about the practicability of the screening and whether they observed increased referral to liaison services. However, the low response rate leading to a small sample of 19 physicians prevented any reasonable analyses. |

| **Table S4.** *Detailed information on included clusters* | | | | |
| --- | --- | --- | --- | --- |
| **Cluster** | **Affiliation** | **T0**  n_IG_/n_CG_ | **T1**  n_IG_/n_CG_ | **T2**  n_IG_/n_CG_ |
| Oncologic ambulance | UCCH | 86/73 | 44/53 | 29/42 |
| Leukemia station | UCCH | 16/31 | 7/17 | 6/15 |
| Private station | UCCH | 56/31 | 28/21 | 19/17 |
| General oncologic station | UCCH | 34/23 | 18/14 | 12/10 |
| Radiation therapy | UCCH | 42/21 | 18/8 | 14/7 |
| “Marienkrankenhaus”, day clinic | External | 18/31 | 6/20 | 3/15 |
| “Marienkrankenhaus”, oncological station | External | 13/12 | 4/1 | 3/1 |
| “Marienkrankenhaus”, private station | External | -/6 | -/5 | -/4 |
| Hemato-oncological practice (“HOPA”) | External | 45/66 | 16/38 | 9/27 |
| Group Practice “Ballindamm” | External | 3/25 | 1/19 | 1/15 |
| “Jerusalem” hospital | External | 20/8 | 10/5 | 8/2 |
| **UCCH,** institutions within the University Cancer Center Hamburg; **External**, institutions within the competence network of the UCCH; **n_IG_** = patients assigned to intervention; **n_CG_** = patients assigned to control condition  **Note:** the cluster “Marienkrankenhaus - private station” only received control condition owing to too few participants | | | | |
